# Creosote Impurities

**Published:** 1883-01

**Authors:** 


### Creosote Impurities.

[Read at the meeting of the American Pharmaceutical Association held at Niagara Falls, 1882, by Prof, P. W. Bedford.

The object of this query can be but one, namely, to inquire whether the wood creosote offered for sale is a pure article, or not; and if not, what is the impurity present?

The relative commercial value of the articles sold as coal tar creosote and wood creosote disposes of the question as to the latter being present in the former article, and we are quite certain that the cheap variety is nothing more or less than a phenol or carbolic acid. Wood creosote, it has been frequently stated, is adulterated with coal tar creosote, or phenol. The object of my experiments has been to prove the identity of wood creosote and its freedom from phenol. The following tests are laid down in various works as conclusive evidence of its purity, and each has been fully tried with the several samples of wood creosote to prove their identity and purity, and also with phenol, sold as commercial creosote or coal tar creosote, and for comparison with mixtures of the two, that even small percentages of admixture might be identified, should such exist in the wood creosote of the market.

The following tests were used:

1. Equal volumes of anhydrous glycerine and wood creosote make a turbid mixture, separating on standing. *Phenol dissolves.* If three volumes of water be added, the separation of the wood creosote is immediate. *Phenol remains in permanent solution.*

2. One volume of wood creosote added to two volumes of glycerine; the former is not dissolved, but separates on standing. *Phenol dissolves.*

3. Three parts of a mixture containing 75 per cent. of glycerine and 25 of water to one part of wood creosote show no increase of volume of glycerine, and wood creosote separates. *Phenol dissolves, and forms a clear mixture.* Were any phenol present in the wood creosote, the increase in the volume of the glycerine solution, if in a graduated tube, would distinctly indicate the percentage of phenol present.

4. Solubility in benzine. Wood creosote entirely soluble. *Phenol is insoluble.*

5. A 1 per cent. solution of wood creosote. Take of this 10 cubic centimeters, add 1 drop of a test solution of ferric chloride; an evanescent blue color is formed, passing quickly into a red color. *Phenol gives a permanent blue color.*

6. Collodion or albumen with an equal bulk of wood creosote makes a perfect mixture without coagulation. *Phenol at once coagulates into a more or less firm mass or clot.*

7. Bromide solution with wood creosote gives a reddish brown precipitate. *Phenol gives a white precipitate.*

All tests enumerated above were repeatedly tried with four samples of wood creosote sold as such ; one a sample of Morson's, one of Merck's, one evidently of German origin, but bearing the label and capsule of an American manufacturer, and one of unknown origin, but sold as beech-wood creosote (German), and each proved to be *pure wood creosote.*

Two samples of commercial creosote which, from the low cost, were known to be of coal tar origin gave the negative tests, showing that they were phenol.

Corroborative experiments were made by mixing 10 to 20 per cent. of phenol with samples of the beechwood creosote, but in every case each of the tests named showed the presence of the phenol.

The writer on other occasions applied single tests (the collodion test) to samples of beechwood creosote that he had an opportunity of procuring small specimens of, and satisfied himself that they were pure. The conclusion is that the wood creosote of the market of the present time is in abundant supply, is of unexceptionable quality, and reasonable in price, so that there is no excuse for the substitution of the phenol commonly sold for it. When it is directed for use for internal administration (the medicinal effect being entirely dissimilar), wood creosote only should be dispensed.

The general sales of creosote by the pharmacist are in small quantities as a toothache remedy, and phenol has the power of coagulating albumen, which effectually relieves the suffering. Wood creosote does not coagulate albumen, and is, therefore, not as serviceable. This is, perhaps, the reason that it has become, in a great measure, supplanted in general sale by the coal tar creosote, to say nothing of the argument of a lower cost.

